# CD44V3, an Alternatively Spliced Form of CD44, Promotes Pancreatic Cancer Progression

**DOI:** 10.3390/ijms232012061

**Published:** 2022-10-11

**Authors:** Hanzhang Zhu, Weijiang Zhou, Yafeng Wan, Jun Lu, Ke Ge, Changku Jia

**Affiliations:** Department of Hepatopancreatobiliary Surgery, Hangzhou First People’s Hospital, The Affiliated Hospital of Medical School of Zhejiang University, Research Center of Diagnosis and Treatment Technology for Hepatocellular Carcinoma of Zhejiang Province, Hangzhou 310006, China

**Keywords:** CD44V3, U2AF1, splicing, pancreatic cancer, stemness

## Abstract

Pancreatic cancer is one of the most lethal malignant tumors. However, the molecular mechanisms responsible for its progression are little known. This study aimed to understand the regulatory role of CD44V3 in pancreatic cancer. A Kaplan–Meier analysis was performed to reveal the correlation between CD44/CD44V3 expression and the prognosis of pancreatic cancer patients. *CD44V3* and *U2AF1* were knocked down using shRNAs. The proliferation, migration, invasion, and stemness of two pancreatic cell lines, BxPC-3 and AsPC-1, were examined. The expression of CD44V3, cancer-associated markers, and the activation of AKT signaling were detected by qRT-PCR and Western blot. Both CD44 and CD44V3 expression levels were associated with a poor prognosis in pancreatic cancer patients. Interestingly, the expression of CD44V3, instead of CD44, was greatly increased in tumor tissues. *CD44V3* knockdown inhibited the proliferation, migration, invasion, and stemness of cancer cells. *CD44V3* splicing was regulated by U2AF1 and downregulation of U2AF1 enhanced CD44V3 expression, which promoted pancreatic cancer progression. CD44V3 is an important cancer-promoting factor, which may serve as a potential candidate for pancreatic cancer intervention.

## 1. Introduction

Pancreatic cancer, one of the most lethal malignant tumors, caused more than 432,000 deaths in 2018 worldwide [1]. The main risk factors for pancreatic cancer include a family history of chronic pancreatitis, smoking, diabetes, age, and some genetic disorders [2,3,4,5]. Although massive efforts have been invested in past decades, surgery, radiation, chemotherapy, and other conventional approaches have showed little effect on the treatment of this malignant tumor [6,7,8,9]. To date, surgical resection provides the best chance of cure. However, more than 80% of patients develop advanced unresectable pancreatic cancer, and less than 5% of patients survive five years after diagnosis [10]. Hence, it is very urgent to understand the biological mechanisms underlying the development and progression of pancreatic cancer.

Cancer stem cells are a subset of the cell population that is able to dictate the heterogeneity, proliferation, metastasis, and multi-drug resistance in tumors [11,12]. The hyaluronic acid receptor CD44 is a cancer stem cell marker involved in multiple regulation of almost all cancer types, such as cell survival, proliferation, motility, and tumor microenvironment remodeling [13,14,15]. In human glioblastoma multiforme, CD44 enhanced the cancer stem cell phenotypes and promoted therapeutic resistance, whose expression level was correlated with a poor survival rate [14]. High CD44 levels have been linked to cell proliferation and tumorigenesis in everything from solid tumors to hematologic malignancies, and siRNA knockdown of CD44 could significantly inhibit tumorigenicity [11]. Furthermore, CD44 is one of the most commonly used cancer stem cell surface markers in sorting different subpopulations of tumor tissues [16,17,18].

The *CD44* gene contains 20 exons, half of which are constant exons expressed in all isoforms, while the other half are variable exons (V1-10) undergoing extensive alternative splicing by spliceosome [19,20]. Alternative splicing can produce many CD44 isoforms that possess various tissue-specific effects during cancer progression [20]. *CD44V3* contains exon V3 that encodes a heparin sulfate site, which allows it to bind heparin sulfate binding growth factors, such as epidermal growth factor (EGF) and fibroblast growth factors (FGFs) [21,22,23]. CD44 V8-10 can stabilize xCT, a cysteine glutamate transporter, and then enhance cystine uptake and intracellular antioxidant synthesis [24]. The inclusion of variant exons of *CD44* is regulated by oncogenic signals, which might contribute to the expression of a large variety of *CD44* variants in advanced stage tumors [25,26].

In the current study, the regulatory roles of CD44 in pancreatic cancer progression and prognosis were investigated. Although CD44 levels correlated with poor prognosis, in comparison with adjacent normal tissues, its expression level was decreased in tumor tissues. However, the expression of CD44V3 was significantly increased in tumor tissues and correlated with the poor prognosis of patients. Functional studies indicated that the elevation of CD44V3 was regulated by U2AF1, an important component of the spliceosome, which then enhanced the proliferation and invasion ability and promoted epithelial-mesenchymal transition (EMT) and stemness of cancer cells [27,28]. The tumor promoting effect of CD44V3 might be mediated by the AKT signaling pathway. This finding suggests that targeting the CD44V3 isoform might pave the way for a novel treatment strategy for pancreatic cancer.

## 2. Results

### 2.1. CD44 Is Correlated with Poor Prognosis of Pancreatic Cancer Patients

**Figure 1:**
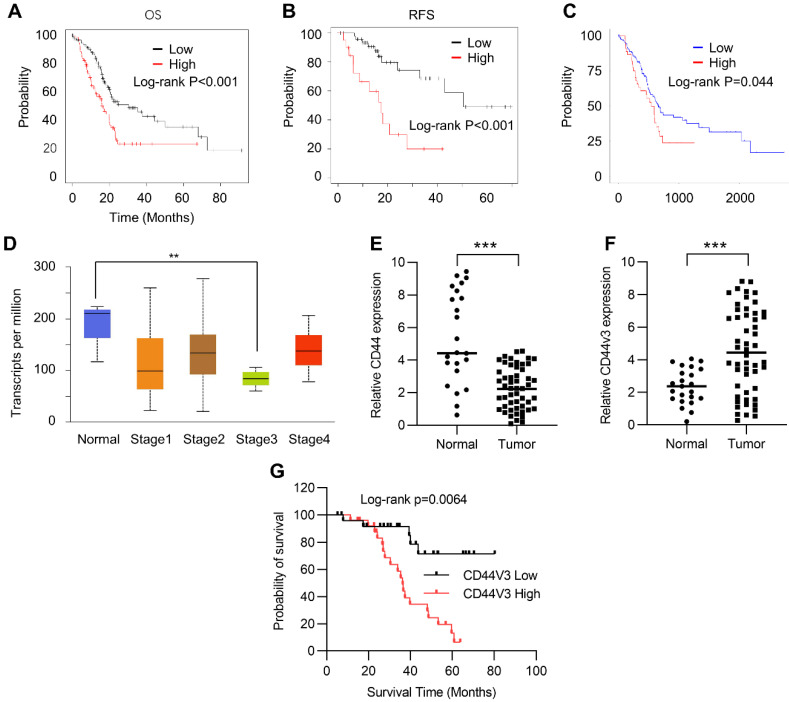
CD44 was correlated with a poor prognosis of pancreatic cancer. (**A**) Kaplan–Meier plots of overall survival in pancreatic cancer patients stratified according to their CD44 levels. The Kaplan–Meier plotter was used to analyze the data. (**B**) Kaplan–Meier plots of release-free survival in pancreatic cancer patients stratified according to their CD44 levels. The Kaplan–Meier plotter was used to analyze the data. (**C**) Kaplan–Meier plots of overall survival in pancreatic cancer patients stratified according to their CD44 levels. The data was analyzed in TCGA dataset from ualcan (http://ualcan.path.uab.edu, accessed on 8 May 2021). (**D**) CD44 levels in normal and pancreatic tumor tissues at various stages. The data was analyzed using the TCGA dataset from ualcan (http://ualcan.path.uab.edu, accessed on 8 May 2021). (**E**) The CD44 levels in normal and pancreatic tumor tissues were analyzed by qPCR. (**F**) The CD44V3 levels in normal and pancreatic tumor tissues were analyzed by qPCR. (**G**) Kaplan–Meier plots of overall survival in pancreatic cancer patients stratified according to their CD44V3 levels. The data are presented as mean ± S.D. ** *p* < 0.01; *** *p* < 0.001.

In order to study the role of CD44 in the progression of pancreatic cancer, we first investigated the correlation between the prognosis and the expression level of CD44 in cancer tissues using the Kaplan–Meier Plotter (Table S1). Kaplan–Meier plots of overall survival (OS) and relapse-free survival (RFS) in pancreatic cancer patients were stratified according to their CD44 levels. The results showed that high CD44 expression levels positively correlated with poor OS and RFS in pancreatic cancer patients (Figure 1A,B). We further analyzed this correlation in the TCGA pancreatic cancer database using Ualcan (http://ualcan.path.uab.edu/, accessed on 8 May 2021) and arrived at a similar result: high CD44 expression predicted poor prognosis (Figure 1C). Next, we examined CD44 expression in normal and different stages of pancreatic tumor tissues using the Kaplan–Meier Plotter. Unexpectedly, CD44 levels were higher in adjacent normal tissues than in pancreatic tumor tissues (Figure 1D). A similar result was observed in normal and pancreatic tumor tissues detected by RT-qPCR (Figure 1E). To explore this contradiction, we examined the expression of CD44V3, which has been demonstrated to have a critical role in regulating the development and occurrence of various tumors, in tumor and normal tissues by RT-qPCR. Our data showed that CD44V3 expression levels were markedly increased in tumor tissues compared to adjacent normal tissues (Figure 1F). Moreover, pancreatic cancer patients with high CD44V3 levels showed poor OS, which showed a more potent correlation than that of CD44 (Figure 1A,G). All this data suggests that CD44V3 might be one of the important players in regulating tumor progression and prognosis of pancreatic cancer patients.

### 2.2. U2AF1 Regulates CD44V3 Splicing

**Figure 2:**
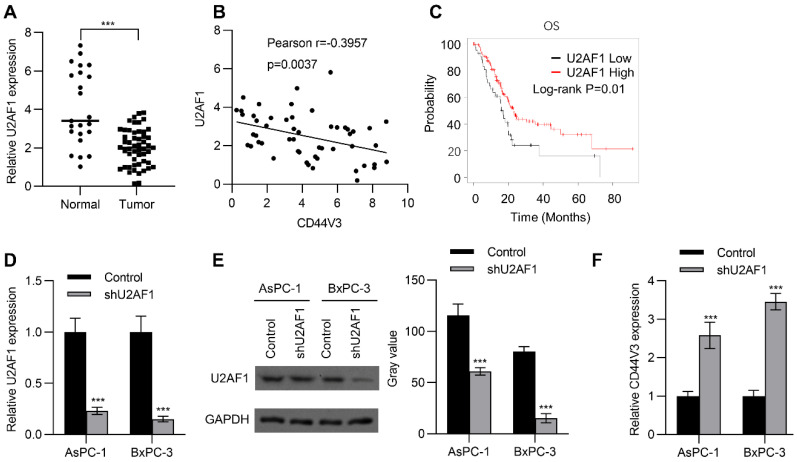
U2AF1 regulates CD44V3 splicing. (**A**) The U2AF1 levels in normal and pancreatic tumor tissues were analyzed by qPCR. (**B**) The correlation between U2AF1 and CD44V3 in pancreatic cancer patients was determined by qPCR. (**C**) Kaplan–Meier plots of overall survival in pancreatic cancer patients stratified according to their U2AF1 levels. The Kaplan–Meier Plotter was used to analyze the data. (**D**,**E**) The U2AF1 levels in AsPC-1 or BxPC-3 cells transfected with U2AF1 shRNA were analyzed by qPCR (**D**) or Western blot (**E**). (**F**) The CD44V3 levels in AsPC-1 or BxPC-3 cells transfected with U2AF1 shRNA were analyzed by qPCR. The data are presented as mean ± S.D. *** *p* < 0.001.

U2AF1 is an RNA splicing complex component of the spliceosome that regulates pre-mRNA splicing [27]. Recent studies have further confirmed that it can regulate alternative splicing of mRNA [28]. Therefore, we wondered whether alternative splicing of CD44V3 is regulated by U2AF1. To this end, we examined the expression of U2AF1 in adjacent normal tissues and pancreatic tumor tissues by RT-qPCR, to explore whether U2AF1 was involved in the regulation of CD44V3 expression. As shown in Figure 2A, U2AF1 levels were significantly reduced in tumors compared to those in normal tissues. Interestingly, U2AF1 expression level was negatively correlated with CD44V3 expression level in pancreatic tumor tissues (Figure 2B). Moreover, Kaplan–Meier analysis showed that higher U2AF1 levels predicted better OS for pancreatic cancer patients (Figure 2C). Since lower U2AF1 expression predicted poorer prognosis, we knocked down U2AF1 in BxPC-3 and AsPC-1 pancreatic cancer cells using shRNA, which significantly reduced both the mRNA and protein levels of U2AF1 (Figure 2D,E), and then measured the expression of CD44V3. As expected, U2AF1 knockdown could significantly increase the expression of CD44V3 in both AsPC-1 and BxPC-3 cells (Figure 2F). The above results indicated that U2AF1 regulated the splicing of CD44 and inhibited the expression of CD44V3.

### 2.3. Downregulation of CD44V3 Inhibits Pancreatic Cancer Cell Proliferation

**Figure 3:**
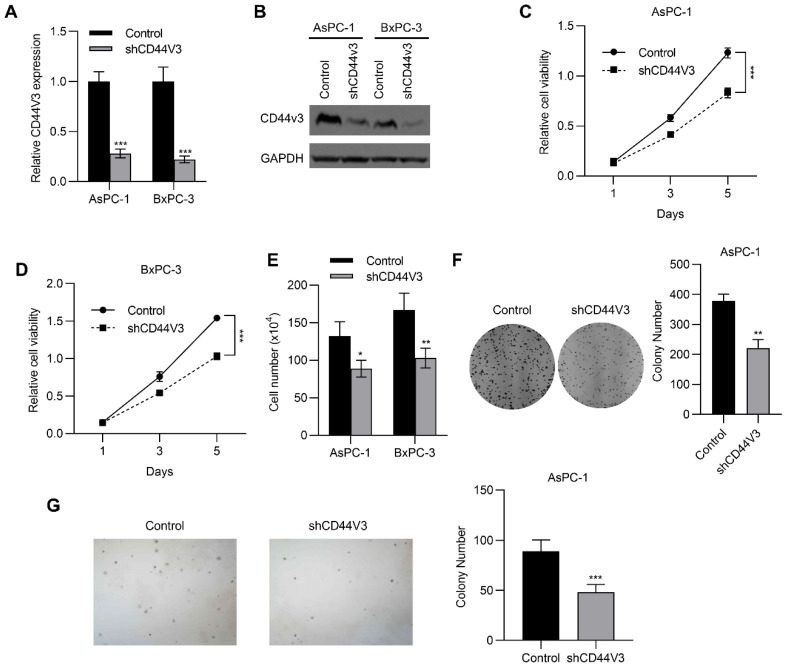
Downregulation of CD44V3 inhibits pancreatic cancer cell proliferation. (**A**,**B**) The CD44V3 levels in AsPC-1 or BxPC-3 cells transfected with CD44V3 shRNA were analyzed by qPCR (**A**) or Western blot (**B**). (**C**,**D**) Cell viability of AsPC-1 (**C**) or BxPC-3 (**D**) cells transfected with CD44V3 shRNA was determined by MTT assay. (**E**) A cell count assay was used to determine the viability of AsPC-1 or BxPC-3 cells transfected with CD44V3 shRNA. (**F**) The foci formation assay was used to determine the viability of AsPC-1 cells transfected with CD44V3 shRNA. (**G**) The cell viability of AsPC-1 cells transfected with CD44V3 shRNA was determined by a soft agar assay. The data are presented as mean ± S.D. * *p* < 0.05; ** *p* < 0.01; *** *p* < 0.001.

To investigate the regulatory role of CD44V3 in pancreatic cancer progression, we knocked down CD44V3 in BxPC-3 and AsPC-1 cells using shRNA and examined the changes in proliferative ability. As shown in Figure 3A,B, both mRNA and protein levels of CD44V3 were markedly reduced. Accordingly, the MTT assay showed that CD44V3 knockdown significantly suppressed proliferation of the two pancreatic cancer cell lines (Figure 3C,D). Cell count assay results indicated that CD44V3 knockdown markedly reduced the viability of both AsPC-1 and BxPC-3 cells (Figure 3E). We performed cell colony formation and soft agar assays to assess the cell viability and anchorage-independent cell growth ability with or without CD44V3. The results showed that knockdown of CD44V3 significantly reduced the colony formation of AsPC-1 cells, while interestingly, the average size of the clone was smaller than that in the control group (Figure 3F,G), indicating that CD44V3 deficiency inhibited the proliferation of pancreatic cancer cells.

### 2.4. Downregulation of CD44V3 Suppresses Pancreatic Cancer Cells Invasion and Stemness

**Figure 4:**
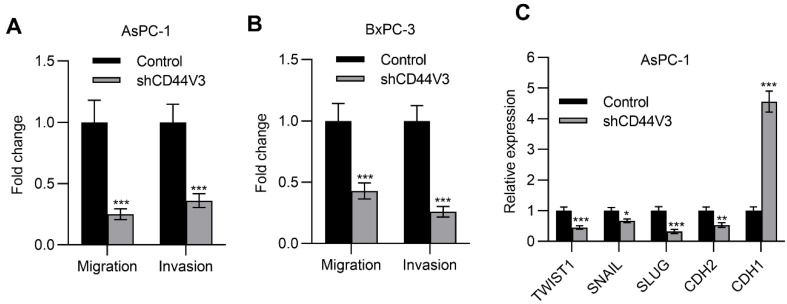
Downregulation of CD44V3 inhibited pancreatic cancer cell invasion. (**A**) Transwell migration and invasion assay of AsPC-1 cell transfected with CD44V3 shRNA. (**B**) Transwell migration and invasion assay of BxPC-3 cell transfected with CD44V3 shRNA. (**C**) A panel of EMT markers in AsPC-1 cells transfected with CD44V3 shRNA was determined by qPCR. The data are presented as mean ± S.D. * *p* < 0.05; ** *p* < 0.01; *** *p* < 0.001.

**Figure 5:**
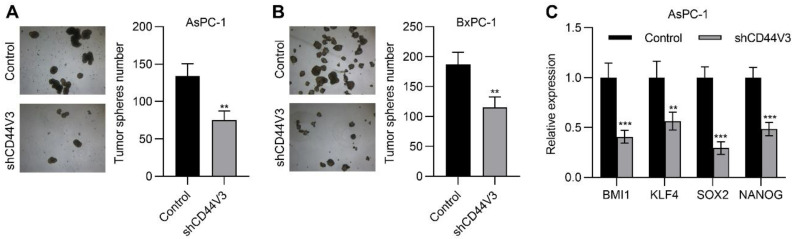
Downregulation of CD44V3 inhibited pancreatic cancer cell stemness. (**A**) Tumor spheres formation assay of AsPC-1 cells transfected with CD44V3 shRNA. (**B**) Tumor sphere formation assay of BxPC-3 cells transfected with CD44V3 shRNA. (**C**) A panel of stem cell markers in AsPC-1 cell transfected with CD44V3 shRNA was determined by qPCR. The data are presented as mean ± S.D. ** *p* < 0.01; *** *p* < 0.001.

Next, we examined the pancreatic cancer cell invasion ability with or without CD44V3 by using transwell migration and invasion assays. The knockdown of CD44V3 greatly suppressed the migration and invasion capacity of AsPC-1 cells (Figure 4A). A similar inhibitory effect of cancer cell invasion was observed in BxPC-3 cells (Figure 4B). Furthermore, we measured a panel of EMT markers in control and CD44V3 knockdown AsPC-1 cells and found that mesenchymal markers (TWIST1, SNAIL, SLUG, and CDH2) were decreased in CD44V3 knockdown cells, whereas the epithelial marker CDH1 was significantly increased (Figure 4C). We further detected the cancer cell stemness ability of CD44V3 knockdown in both AsPC-1 and BxPC-3 cells through a tumor sphere formation assay. The results showed that knockdown of CD44V3 could significantly reduce the average size of AsPC-1 and BxPC-3 spheres, indicating that CD44V3 deficiency suppresses the balloon-forming ability of pancreatic cancer cells (Figure 5A,B). Stem cell markers (BMI1, KLF4, SOX2, and NANOG) in AsPC-1 cells were measured by RT-qPCR, all of which were significantly decreased in CD44V3 knockdown cells compared to the control group (Figure 5C). All this data indicated that CD44V3 was able to promote cellular migration, invasion, and stemness of pancreatic cancer cells.

### 2.5. CD44V3 Increases the AKT Signaling Pathway

**Figure 6:**
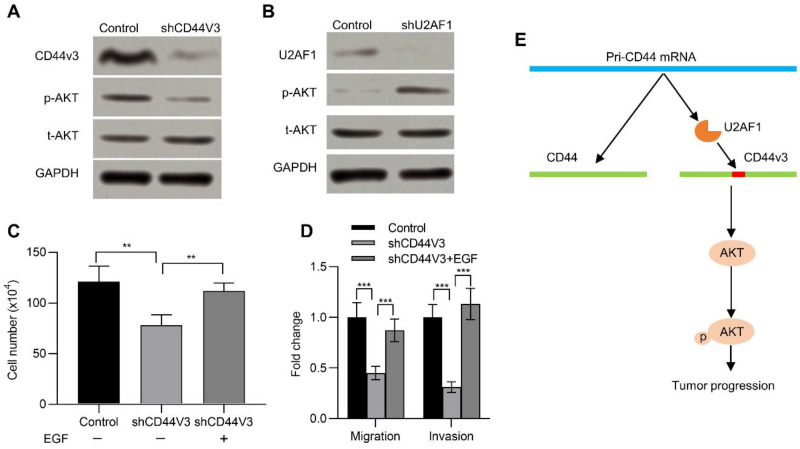
CD44V3 increased the AKT signaling pathway. (**A**) The p-AKT and t-AKT levels in AsPC-1 cells transfected with CD44V3 shRNA were determined by Western blot. (**B**) The p-AKT and t-AKT levels in AsPC-1 cells transfected with U2AF1 shRNA were determined by Western blot. (**C**) Cell count assay of AsPC-1 cells transfected with CD44V3 shRNA and treated with or without recombinant EGF. (**D**) Transwell migration and invasion assay of the AsPC-1 cells transfected with CD44V3 shRNA and treated with or without recombinant EGF. (**E**) A graphical abstract of the role of CD44V3 in tumor progression. The data are presented as mean ± S.D. ** *p* < 0.01; *** *p* < 0.001.

The AKT pathway is frequently activated in many kinds of human cancers and has a critical impact in neoplastic transformation [29,30]. Previous studies suggested that CD44 activated the AKT pathway [31]. We therefore wanted to determine if CD44v3 could also activate the AKT pathway. To this end, we examined its activation in pancreatic cancer cells with or without CD44V3. Knockdown of CD44V3 reduced the p-AKT level in AsPC-1 cells compared to that of control (Figure 6A). On the contrary, knockdown of U2AF1, which promoted the expression of CD44V3, could significantly increase the expression of p-AKT (Figure 6B). In addition, EGF, which activates the AKT signaling pathway and enhances tumor growth, invasion, and metastasis [32], recovered the CD44V3 knockdown-inhibited cell viability (Figure 6C). Moreover, EGF treatment could rescue the invasion and migration ability of pancreatic cancer cells, which was inhibited in CD44V3 knockdown pancreatic cancer cells (Figure 6D). Taken together, U2AF1 mediated the splicing of CD44V3. The upregulation of CD44V3 could activate the AKT signaling pathway, which eventually promoted pancreatic tumor progression (Figure 6E).

## 3. Discussion

Cancer stem cells contribute to multiple tumor malignancies through their self-renewal and differentiation capacity, including heterogeneity, metastasis, recurrence, and therapeutic resistance. Therefore, cancer stem cells are considered important targets for cancer treatment. Cumulative evidence indicates CD44 is not only a cancer stem cell marker but also a critical regulator of cancer stemness [33,34,35]. Here, we studied the function of CD44 in pancreatic cancer progression and prognosis and found that the expression of one splicing variant, CD44V3, instead of CD44, was significantly increased in tumor tissues, and its level correlated with poor prognosis. Mechanistically, CD44V3 expression was downregulated by splicing factor U2AF1, an important component of the spliceosome, and the expression of U2AF1 was reduced in pancreatic cancer cells and tissues [27,28]. Elevated CD44V3 enhanced cell proliferation and invasion and promoted tumor migration and stemness of pancreatic cancer cells, which might be partially mediated through the AKT signaling pathway.

Both standard and variable CD44 isoforms are widely expressed in most human normal and carcinomatous tissues [19,20,36]. Alternative splicing of CD44 can generate more than 1000 isoforms theoretically, ranging from 85 to 250 kDa [20]. CD44 isoforms (CD44V) play important roles in cancer, including cancer cell stemness, metastasis, and tumor initiation. For example, in comparison with adjacent normal tissues, CD44V4-5 is highly expressed in breast cancer tissues [37]. In colorectal carcinoma, elevated CD44V6 promoted cancer progression to later stages through activating the Stat6 signaling pathway [38,39]. Knockdown of CD44V4-7 blocks tumor cell matrix crosstalk and reduces the metastatic potential of rat ASML cells [40]. In line with the above findings on CD44V, we demonstrated that CD44V3 expression level was markedly increased in pancreatic cancer tissues and was closely correlated with poor OS and RFS in 52 patients. Knockdown of CD44V3 could inhibit cell proliferation, invasion, and migration of pancreatic cancer cells. Furthermore, CD44V3 knockdown significantly suppressed the expression of multiple stemness markers, including BMI1, KLF4, SOX2, and NANOG [41], indicating that, in accordance with CD44V, CD44V3 possesses stemness-maintaining ability and then promotes pancreatic cancer migration and invasion.

The alternative splicing of CD44 is being revealed gradually [42]. Recent studies have indicated the relationship between autocrine growth factor signaling and alternative splicing regulated by epithelial splicing regulatory proteins 1 and 2 (ESRP1 and ESRP2) [43]. Studies have also showed that ESRP1 and ESRP2 are involved in the splicing regulation of CD44V6-10 and V8-10 [43,44]. However, overexpression of ESRP1 cannot induce a differential CD44V splicing pattern in breast cancer cells [45], which suggests that ESRP1 is not the major regulator of CD44V splicing. In this study, we examined another splicing factor, U2AF1, in pancreatic cancer progression and found it was significantly downregulated in pancreatic tumor tissues. In contrast to CD44V3, U2AF1 expression level was correlated with better prognosis of pancreatic cancer patients. Moreover, knockdown of U2AF1 could significantly increase the expression level of CD44V3, which indicates that the splicing and expression of CD44V3 are regulated by U2AF1 in pancreatic cancer cells. In future studies, it would be interesting to look into methods to modulate the activation of the U2AF1 and/or CD44V3 axis for pancreatic cancer treatment.

## 4. Materials and Methods

### 4.1. Patients

Pancreatic cancer tissues and the paired adjacent non-tumor tissues were collected from 52 patients who received surgical resection of pancreatic tumors at the Hangzhou First People’s Hospital. Table S1 contains information on cancer type, histology subtype, and tumor stage. The excised tissue samples were frozen by liquid nitrogen and stored at −80 °C. For gene expression analysis, total RNA was extracted from tumor and adjacent non-tumor tissues. All the patients provided informed consent following a protocol approved by the ethics board of Hangzhou First People’s Hospital (#2019-61-01, 25 February 2019), and this study was conducted in accordance with the ethical standards formulated in Hangzhou First People’s Hospital.

### 4.2. Cell Culture

Two human pancreatic cancer cell lines, AsPC-1 (CRL-1682™) and BxPC-3 (CRL-1687™), were obtained from ATCC (Manassas, VA, USA). Cells were cultured in RPMI-1640 medium (Thermo Fisher Scientific, Waltham, MA, USA) containing 10% fetal bovine serum (Gibco, Grand Island, NY, USA), 100 μg/mL of streptomycin, 100 IU/mL of penicillin, and 2 mM L-glutamine. Cells were placed in the humidified incubator with 5% CO_2_ at 37 °C. Human epidermal growth factor (EGF) was purchased from Sigma-Aldrich (St. Louis, MO, USA). AsPC-1 cells with or without CD44V3 were treated by recombinant EGF (50 nmol/L) for 24 h, then transwell migration and invasion assays were performed.

### 4.3. Gene Knockdown in Pancreatic Cancer Cells

The expression of *CD44V3* and *U2AF1* in pancreatic cancer cells was inhibited through shRNA-mediated gene knockdown. Control, *CD44V3*, and *U2AF1* shRNAs were ordered from GenePharma (Shanghai, China). The shRNA sequence for *CD44V3*: GATCCGAAGATAAAGACCATCCAATTCAAGAGATTGGATGGTCTTTATCTTCTTTTTTGGAAA. The shRNA sequence for *U2AF1*: CCGGGAAAGTGTTGTAGTTGATTGACTCGAGTCAATCAACTACAACACTTTCTTTTT. To knockdown the target genes, AsPC-1 and BxPC-3 cells were transfected with control shRNA, *CD44V3* shRNA (shCD44V3), and *U2AF1* shRNA (shU2AF1) constructs, respectively, by using the Lipofectamine 3000 reagent (Thermo Fisher Scientific) following the manufacturer’s instructions. The knockdown of CD44V3 and U2AF1 was verified by RT-qPCR.

### 4.4. qRT-PCR

Total RNA of normal and pancreatic tumor tissues and treated pancreatic cancer cells was extracted by using the TRIzol™ Reagent (Invitrogen, Waltham, MA, USA), according to the manufacturer’s instructions. The first-strand cDNA was synthesized using the M-MLV Reverse Transcriptase (Beyotime, Shanghai, China). RT-qPCR was conducted using the CFX Opus Real-Time PCR System (Bio-Rad Laboratories, Inc., Hercules, CA, USA). The relative expression of target genes was normalized to *GAPDH* and calculated by using the 2^-ΔΔCT^ method. The specific qPCR primers for *CD44V3* are: forward-TTAGGGTGCTACCAGTAC; reverse-GTCTCTGGTGCTGGAGATAA. The qPCR primers for *CD44* are: forward-CCAGTCATAGTATAACGCTTC; reverse-TCTTTATCTTCTTCCAAGCCT; *U2AF1*: forward-TTTCAAAATTGGAGCATGTCG; reverse- TGCATCTCCACATCGCTCA; and *GAPDH*: forward-TGACTTCAACAGCGACACC; reverse-TTGCTGTAGCCAAATTCGTT.

### 4.5. Western Blot

Control and CD44V3 or U2AF1 knockdown AsPC-1 cells were lysed using the lysis buffer (Beyotime) with a protease inhibitor cocktail (Promega, Madison, WI, USA). Target protein expression was determined by Western blot as previously described [31]. The primary antibodies were ordered from Cell Signaling (Danvers, MA, USA), and the detailed information was listed below: phospho-AKT (p-AKT, Ser473) (#9271, 1:2000 dilution), total-AKT (#4691, 1:2000 dilution), and GAPDH (#5174, 1:3000 dilution).

### 4.6. Cell Viability

The cell viability of AsPC-1 and BxPC-3 cells with or without CD44V3 was determined by cell count assay and MTT assay. In brief, control and CD44V3 knockdown cells were placed into a 6-well plate at a density of 1 × 10^5^ cells per well, and the total cell numbers of each well were counted three times on day 3 using the Beckman Coulter Vi-Cell (Brea, CA, USA). For the MTT assay, control and CD44V3 knockdown cells were seeded in a 96-well plate at a density of 1 × 10^3^ cells per well. Cell viability on days 1, 3, and 5 was measured using MTT cell proliferation and cytotoxicity assay kits (Beyotime, Shanghai, China).

### 4.7. Cell Colony Formation and Soft Agar Colony Formation Assays

Control and CD44V3 knockdown AsPC-1 cells were seeded into 35 mm plates (1 × 10^3^ cells per well) and cultured with fresh media every 3 days. When they reached appropriate confluency, cells were stained with crystal violet (0.2%)/formaline (10%), and the signal of crystal violet was quantified at 700 nm using the GloMax^®^ Discover Microplate Reader (Promega, Madison, WI, USA). The anchorage-independent growth ability of control and CD44V3 knockdown AsPC-1 cells was determined by a soft agar colony formation assay as previously described [46].

### 4.8. Migration and Invasion Assay

The invasion and migration of control and CD44V3 knockdown AsPC-1 cells were measured by using a 6.5 mm transwell chamber (8 μm pore, BD, San Jose, CA, USA). Briefly, serum-free medium was used to suspend cells (3 × 10^4^ cells per well), which were subsequently cultured in the upper chamber. Complete culture medium was then added into the lower chamber. After 24 h of culture, the migrated/invaded cells (lower chamber) were fixed with 4% paraformaldehyde, and then stained with crystal violet (0.1 mg/mL) and counted using a Nikon microscope. Three visual fields were counted randomly for each well, and the average was presented.

### 4.9. Tumor Sphere Formation Assay

Control and CD44V3 knockdown AsPC-1 or BxPC-3 cells were suspended at a density of 5 × 10^4^ cells/mL in complete culture medium and seeded into 6-well plates (2.5 mL per plate) coated with 1.2% polyhema. Fresh culture medium was changed every 3 days, and cells were passaged every week. As previously described, tumor spheres were measured and counted using Zeiss Axiovision software (Carl Zeiss, Jena, Germany) [47].

### 4.10. Statistical Analysis

The correlation between pancreatic cancer progression and gene expression of CD44 or U2AF1 was analyzed by using the Kaplan–Meier Plotter. Statistical analyses were performed by using the GraphPad Prism. Two- or one-way analysis of variance (ANOVA) and Student’s t-test were used to analyze the differences between groups. The data were presented as mean ± standard deviation (SD), * *p* < 0.05, ** *p* < 0.01, and *** *p* < 0.001 compared to the control group.

## 5. Conclusions

This study highlights CD44V3 as an important cancer-promoting factor in pancreatic cancer progression, supporting its role as a potential therapeutic target for pancreatic cancer intervention.

## Data Availability

Data could be obtained upon reasonable request to the corresponding author.

## Supplementary Materials

Supplementary materials can be found at https://www.mdpi.com/article/10.3390/ijms232012061/s1.
