# Supplementary material for: CD44V3, an Alternatively Spliced Form of CD44, Promotes Pancreatic Cancer Progression
Source: Int J Mol Sci. 2022 Oct 11;23(20):12061. doi: 10.3390/ijms232012061 (PMC9603666; doi:10.3390/ijms232012061)
Supplement: Supplementary file 1 [file ijms-23-12061-s001.zip › ijms-1912107-supplementary.pdf]

Supplementary materials

Table S1. Clinical characteristics of the participants.

Patient	Age	Gender	Cancer Type	Histologic Subtype	Tumor Stage
1	78	Male	Pancreatic Cancer	Pancreatic Ductal Adenocarcinoma	3
2	80	Male	Pancreatic Cancer	Pancreatic Ductal Adenocarcinoma	4
3	51	Male	Pancreatic Cancer	Pancreatic Ductal Adenocarcinoma	2
4	47	Female	Pancreatic Cancer	Pancreatic Ductal Adenocarcinoma	2
5	78	Male	Pancreatic Cancer	Pancreatic Ductal Adenocarcinoma	2
6	70	Female	Pancreatic Cancer	Pancreatic Ductal Adenocarcinoma	3
7	65	Female	Pancreatic Cancer	Pancreatic Ductal Adenocarcinoma	2
8	63	Male	Pancreatic Cancer	Pancreatic Ductal Adenocarcinoma	3
9	60	Female	Pancreatic Cancer	Pancreatic Ductal Adenocarcinoma	2
10	65	Male	Pancreatic Cancer	Pancreatic Ductal Adenocarcinoma	2
11	64	Female	Pancreatic Cancer	Pancreatic Ductal Adenocarcinoma	3
12	62	Male	Pancreatic Cancer	Pancreatic Ductal Adenocarcinoma	2
13	49	Male	Pancreatic Cancer	Pancreatic Ductal Adenocarcinoma	3
14	70	Female	Pancreatic Cancer	Pancreatic Ductal Adenocarcinoma	1
15	69	Male	Pancreatic Cancer	Pancreatic Ductal Adenocarcinoma	2
16	85	Female	Pancreatic Cancer	Pancreatic Ductal Adenocarcinoma	3
17	44	Male	Pancreatic Cancer	Pancreatic Ductal Adenocarcinoma	2
18	71	Male	Pancreatic Cancer	Pancreatic Ductal Adenocarcinoma	3
19	72	Female	Pancreatic Cancer	Pancreatic Ductal Adenocarcinoma	2
20	53	Female	Pancreatic Cancer	Pancreatic Ductal Adenocarcinoma	4
21	78	Male	Pancreatic Cancer	Pancreatic Ductal Adenocarcinoma	3
22	59	Female	Pancreatic Cancer	Pancreatic Ductal Adenocarcinoma	3
23	48	Female	Pancreatic Cancer	Pancreatic Ductal Adenocarcinoma	3
24	51	Male	Pancreatic Cancer	Pancreatic Ductal Adenocarcinoma	3
25	67	Male	Pancreatic Cancer	Pancreatic Ductal Adenocarcinoma	3
26	53	Male	Pancreatic Cancer	Pancreatic Ductal Adenocarcinoma	3
27	80	Female	Pancreatic Cancer	Pancreatic Ductal Adenocarcinoma	3
28	76	Female	Pancreatic Cancer	Pancreatic Ductal Adenocarcinoma	2
29	60	Female	Pancreatic Cancer	Pancreatic Ductal Adenocarcinoma	3
30	48	Female	Pancreatic Cancer	Pancreatic Ductal Adenocarcinoma	2
31	61	Female	Pancreatic Cancer	Pancreatic Ductal Adenocarcinoma	3
32	64	Male	Pancreatic Cancer	Pancreatic Ductal Adenocarcinoma	3
33	85	Female	Pancreatic Cancer	Pancreatic Ductal Adenocarcinoma	2
34	61	Female	Pancreatic Cancer	Pancreatic Ductal Adenocarcinoma	3
35	54	Male	Pancreatic Cancer	Pancreatic Ductal Adenocarcinoma	2
36	52	Male	Pancreatic Cancer	Pancreatic Ductal Adenocarcinoma	3
37	86	Male	Pancreatic Cancer	Pancreatic Ductal Adenocarcinoma	4
38	67	Female	Pancreatic Cancer	Pancreatic Ductal Adenocarcinoma	2
39	82	Female	Pancreatic Cancer	Pancreatic Ductal Adenocarcinoma	2

40	64	Male	Pancreatic Cancer	Pancreatic Ductal Adenocarcinoma	2
41	45	Male	Pancreatic Cancer	Pancreatic Ductal Adenocarcinoma	2
42	63	Female	Pancreatic Cancer	Pancreatic Ductal Adenocarcinoma	4
43	54	Female	Pancreatic Cancer	Pancreatic Ductal Adenocarcinoma	3
44	82	Male	Pancreatic Cancer	Pancreatic Ductal Adenocarcinoma	4
45	76	Male	Pancreatic Cancer	Pancreatic Ductal Adenocarcinoma	3
46	41	Female	Pancreatic Cancer	Pancreatic Ductal Adenocarcinoma	2
47	50	Male	Pancreatic Cancer	Pancreatic Ductal Adenocarcinoma	2
48	64	Male	Pancreatic Cancer	Pancreatic Ductal Adenocarcinoma	2
49	73	Female	Pancreatic Cancer	Pancreatic Ductal Adenocarcinoma	2
50	64	Male	Pancreatic Cancer	Pancreatic Ductal Adenocarcinoma	1
51	77	Female	Pancreatic Cancer	Pancreatic Ductal Adenocarcinoma	3
52	83	Male	Pancreatic Cancer	Pancreatic Ductal Adenocarcinoma	2
